# The Protective Effect of Cordycepin on D-Galactosamine/Lipopolysaccharide-Induced Acute Liver Injury

**DOI:** 10.1155/2017/3946706

**Published:** 2017-03-28

**Authors:** Jin Li, Liping Zhong, Haibo Zhu, Fengzhong Wang

**Affiliations:** ^1^Institute of Food Science and Technology, Chinese Academy of Agricultural Sciences (CAAS), Beijing 100193, China; ^2^Life Science College of Tarim University, Xinjiang 843300, China; ^3^State Key Laboratory for Bioactive Substances and Functions of Natural Medicines, Beijing Key Laboratory of New Drug Mechanisms and Pharmacological Evaluation Study, Institute of Materia Medica, Chinese Academy of Medical Sciences & Peking Union Medical College, Beijing 100050, China

## Abstract

As the major active ingredient of *Cordyceps militaris*, cordycepin (3′-deoxyadenosine) has been well documented to alleviate inflammation and oxidative stress both in vitro and in vivo. To explore the potential protective effect of cordycepin in fulminant hepatic failure, mice were pretreated with cordycepin for 3 weeks followed by D-galactosamine (GalN)/lipopolysaccharide (LPS) injection. Then we found cordycepin (200 mg/kg) administration elevated survival rate, improved liver function, and suppressed hepatocyte apoptosis and necrosis in mice with severe hepatic damage by GalN/LPS treatment. Further, cordycepin inhibited hepatic neutrophil and macrophage infiltration and prevented proinflammatory cytokine production possibly through suppressing TLR4 and NF-*κ*B signaling transduction. The blockade of reactive oxygen species (ROS) and lipid peroxidation production by cordycepin was associated with the decrease of NAD(P)H oxidase (NOX) activity. Besides, cordycepin significantly prevented excessive autophagy induced by GalN/LPS in the liver. These data suggested that cordycepin could be a promising therapeutic agent for GalN/LPS-induced hepatotoxicity.

## 1. Introduction

Fulminant hepatic failure is an unusual but dreaded disease with high mortality, characterized by hepatic dysfunction, coagulation disorder, and later multiple organ failure. D-galactosamine (GalN) and lipopolysaccharide- (LPS-) induced acute liver injury is one of the most commonly used murine model resembling hepatitis virus invasion. LPS is considered as the primary inducer to liver injury, while GalN deteriorates the extent of hepatocyte necrosis by inhibiting RNA and protein synthesis [1]. Recent pharmacological researches demonstrated that LPS treatment combined with GalN promotes proinflammatory cytokine generation in hepatic macrophages, including interleukin (IL)-6, IL-1*β*, and tumor necrosis factor-*α* (TNF-*α*), which triggers caspase cascade and bring death to hepatocytes [2]. Concretely, TNF-*α* binds to its receptor (TNFR-1), recruiting and cleaving caspase-8 through Fas-associated protein with death domain (FADD). Activated caspase-8 truncates BH3-only BID protein and thus causes cytochrome c released from mitochondria, cleaving and activating caspase-9. Both caspase-8 and caspase-9 contribute to caspase-3 activation and finally result in cell apoptosis [3]. Subsequently, activated macrophages and neutrophils produce massive reactive oxygen species (ROS) through NAD(P)H oxidase (NOX), which accelerates hepatocyte necrosis in further [4].

Cordycepin, also known as 3′-deoxyadenosine, is one of the main active constituents of the traditional herbal medicine *Cordyceps militaris*, with a broad spectrum of biological activities, including initially identified antiviral, antifungal, and antitumorigenic activities [5–7]. It was later found that cordycepin possesses anti-inflammatory, antioxidative, antithrombotic, and antiadipogenetic activity as well [8–11]. In LPS-stimulated macrophages, pretreatment with cordycepin suppressed TNF-*α* expression and stimulated the production of anti-inflammatory cytokines IL-10 and NO [12, 13]. In LPS-induced human epithelial cells, cordycepin effectively decreased vascular adhesion molecule-1 (VCAM-1) expression and prevented neutrophil infiltration [14]. In high-fat diet or alcohol-induced hepatotoxicity, cordycepin exerted beneficial effects on damaged livers [15, 16]. However, whether cordycepin can prevent acute liver injury caused by GalN/LPS remains unknown. In the present study, we investigated the protective effects and preliminary molecular mechanism of cordycepin on GalN/LPS-induced fulminant hepatic failure. Specifically, cordycepin prolonged survival time, improved liver function, and inhibited hepatocyte apoptosis and necrosis. Notably, cordycepin alleviated inflammatory damage in the liver via suppressing TLR4 expression, inactivating NF*κ*B pathway, and thus decreasing circulating TNF-*α* level. It attenuated oxidative injury possibly by reducing NOX activity and inhibiting ROS production. Besides, we also found that cordycepin could decrease excessive levels of autolysosomes in the liver induced by GalN/LPS challenge.

## 2. Materials and Methods

### 2.1. Treatment of Animals

Male C57Bl/6J mice (18–22 g) were purchased from Charles River Corporation (Beijing, China), given access to food and water ad libitum. All the experiments confirmed to the guidelines from the Animal Care and Committee of the Institute of Materia Medica, Chinese Academy of Medical Sciences & Peking Union Medical College (Beijing, China). Mice were given oral gavage of cordycepin (50 and 200 mg/kg; purity > 98%; Boyle Chemical Corporation, Shanghai, China) dissolved in 0.5% carboxyl methylcellulose sodium salt (CMC, vehicle) daily for consecutive 3 weeks. The corresponding control mice were administrated with an equivalent volume of vehicle. On the last day, the mice were injected intraperitoneally with GalN (700 mg/kg; J&K Scientific, Beijing, China) and LPS (15 *μ*g/kg; *Escherichia coli*, O111:B4; Sigma-Aldrich, St. Louis, USA) dissolved in phosphate-buffered saline (PBS) buffer at 1 h after cordycepin or vehicle administration. For survival experiment, forty mice were randomly assigned into the following groups (each group, *n* = 10): (a) 200 mg/kg cordycepin-treated control group, (b) vehicle-treated GalN/LPS group, (c) 50 mg/kg cordycepin-treated GalN/LPS group, and (d) 200 mg/kg cordycepin-treated GalN/LPS group. The survival rate of each group was monitored for 24 h. For biochemical analysis, forty mice were randomly divided into four experimental groups (each group, *n* = 10): (a) vehicle-treated control group, (b) 200 mg/kg cordycepin-treated control group, (c) vehicle-treated GalN/LPS group, and (d) 200 mg/kg cordycepin-treated GalN/LPS group. Blood and liver samples were harvested at 5 h after GalN/LPS injection.

### 2.2. Analysis of Liver Enzymes and Serum Cytokine Levels

The blood samples were drawn from the orbit, and serum were separated after two hours at room temperature. Alanine aminotransferase (ALT) and aspartate aminotransferase (AST) activities were analyzed using commercial kits from Nanjing Jiancheng Bioengineering Institute (Nanjing, China). Circulating levels of cytokines TNF-*α*, IL-6, IL-1*β*, and necrotic marker high mobility group box 1 (HMGB1) were quantified using enzyme-linked immunosorbent assay (ELISA) kits according to the manufacturer's instructions (Cloud-Clone Corporation; Houston, USA).

### 2.3. Analysis of Histopathology

The liver samples were frozen in OCT embedding medium, and 6 *μ*m thick sections were prepared. Then the slices were stained with hematoxylin-eosin, and the histological changes were evaluated in nonconsecutive, randomly chosen histological fields. Necrotic areas were given in [%] of liver area (five sections in each group) [17]. Apoptotic cells were detected by the terminal deoxynucleotidyl transferase-mediated dUTP nick-labeling (TUNEL) method using an in situ apoptosis detection kit (TaKaRa Co., Shiga, Japan). The number of TUNEL-positive cells was recorded. At least three tissue sections in each group were analyzed.

### 2.4. Immunohistochemistry

After treatment with hydrogen peroxide, frozen sections were incubated with rabbit anti-F4/80 antibody (diluted at 1 : 200, catalogue number: sc-26643, Santa Cruz Biotechnology, Dallas, USA) for 1 h at 37°C. The sections were then incubated in secondary antibody following three washes (catalogue number: SAP-9100, Sino Biological, Beijing, China). Prior to counterstaining with hematoxylin, these sections were colored using diaminobenzidine (DAB) kit. Negative controls were established using rabbit IgG instead of primary antibodies. At least three tissue sections in each group were analyzed.

### 2.5. Analysis of Hepatic Lipid Peroxidation and NOX Activity

Liver tissues were homogenized in a saline buffer to prepare a 1/10 homogenate. The levels of malondialdehyde (MDA), glutathione (GSH), myeloperoxidase (MPO) activity, and NOX activity were measured using detection kits according to the manufacturer's instructions (Nanjing Jiancheng Bioengineering Institute, Nanjing, China). Final results were normalized by the total protein concentration of indicated samples.

### 2.6. Reactive Oxygen Species (ROS) Assay

The hepatic ROS content was determined as described previously [18]. Briefly, freshly prepared liver homogenate was incubated with 2′,7′-dichlorofluorescin diacetate (DCFH-DA) (Sigma-Aldrich, St. Louis, USA) at a final concentration of 10 *μ*mol/L for 15 min at 37 °C. After centrifugation, the pellet was resuspended in potassium phosphate buffer (pH 7.4) and incubated for 60 min at 37 °C. Fluorescence was measured at wavelengths of 488 nm for excitation and 525 nm for emission. The amount of 2′,7′-dichlorofluorescein (DCF) was corresponded to a standard DCF curve. Final results were normalized by the total protein concentration of indicated samples.

Frozen liver sections were stained with dihydroethidium (DHE, Ex: Em = 510/595 nm) and observed under a fluorescence microscope (BX51W1; Olympus, Japan). All images were obtained by Volocity software (6.3; PerkinElmer, USA) with a magnification of 400x. At least three tissue sections in each group were analyzed.

### 2.7. Quantitative Real-Time Polymerase Chain Reaction (qRT-PCR) Analysis

Total RNA was extracted from liver tissues by Trizol Reagent (Sigma-Aldrich, St. Louis, USA). cDNAs were synthesized using reverse transcription kit (Toyobo, Shanghai, China) and then was amplified in polymerase chain reactions (PCRs) with SYBR Green Supermix kit (Toyobo, Shanghai, China). The sequences of primers are as follows: mouse TNF-*α*, forward 5′-GCCTCTTCTCATTCCTGCTTGT-3′, reverse 5′-TTGAGATCCATGCCGTTG-3′; mouse IL-1*β*, forward 5′-TTGACGGACCCCAAAAGAT-3′, reverse 5′-GATGATCTGAGTGTGAGGGTCTG-3′; mouse IL-6, forward 5′-GCTACCAAACTGGATATAATCAGGA-3′, reverse 5′-CCAGGTAGCTATGGTACTCCAGAA-3′; and mouse GAPDH, forward 5′-GCCTGGAGAAACCTGCCAAGTAT-3′, reverse 5′-GATGCCTGCTTCACCACCTTC-3′. The reactions were performed on Applied Biosystems Prism 7900HT. GAPDH served as internal normalization control.

### 2.8. Western Blot Analysis

Proteins were prepared from liver tissues in RIPA lysis buffer with protease inhibitors and phosphatase inhibitors cocktail (Roche, Switzerland). Then SDS-PAGE was conducted, and bands were transferred to PVDF membrane, incubating with primary antibodies against toll-like receptor 4 (TLR4, diluted at 1 : 1000, catalog number: ab45104, Abcam, Cambridge, UK), microtubule-associated protein light chain 3 (LC3, diluted at 1 : 1000, catalog number: ab48394, Abcam, Cambridge, UK), p62 (diluted at 1 : 1000, catalog number: ab56416, Abcam, Cambridge, UK), phosphor-IκB (diluted at 1 : 1000, catalog number: ab12135, Abcam, Cambridge, UK), I*κ*B (diluted at 1 : 1000, catalog number: 4814, Cell Signaling Technology, Boston, USA), caspase-3, caspase-8, caspase-9, and poly-(ADP-ribose) polymerase (PARP) (diluted at 1 : 1000, catalog numbers: 9665, 4790, 9508, and 9532, Cell Signaling Technology, Boston, USA). Corresponding secondary antibodies (diluted at 1 : 5000, catalog numbers: ZDR-5306 or ZDR-5307, Sino Biological, Beijing, China) were added, and bands were visualized with enhanced chemiluminescence reagents (Thermo Fisher Scientific, Waltham, USA). Signals were normalized to that of *β*-actin (diluted at 1 : 5000, catalog number: 4967, Cell Signaling Technology, Boston, USA). Five replicates were used for statistical analysis.

### 2.9. Electron Microscopic (EM) Analysis

Liver tissues were fixed in 2.5% glutaraldehyde followed by 1% OsO_4_. After dehydration, thin sections were stained with uranyl acetate and lead citrate. Images were acquired by New Bio-TEM H-7500 (Hitachi, Tokyo, Japan).

### 2.10. Data Analysis

Survival data were analyzed by the Kaplan-Meier curve and log-rank test. All other data were analyzed by one-way analysis of variance (ANOVA), and the Bonferroni test was used for post hoc comparisons. The differences between each group were considered statistically significant at a *p*value < 0.05. The results are presented as mean ± SEM.

## 3. Results

### 3.1. Pretreatment of Cordycepin Attenuated Acute Hepatic Damage Induced by GalN/LPS

**Figure 1:**
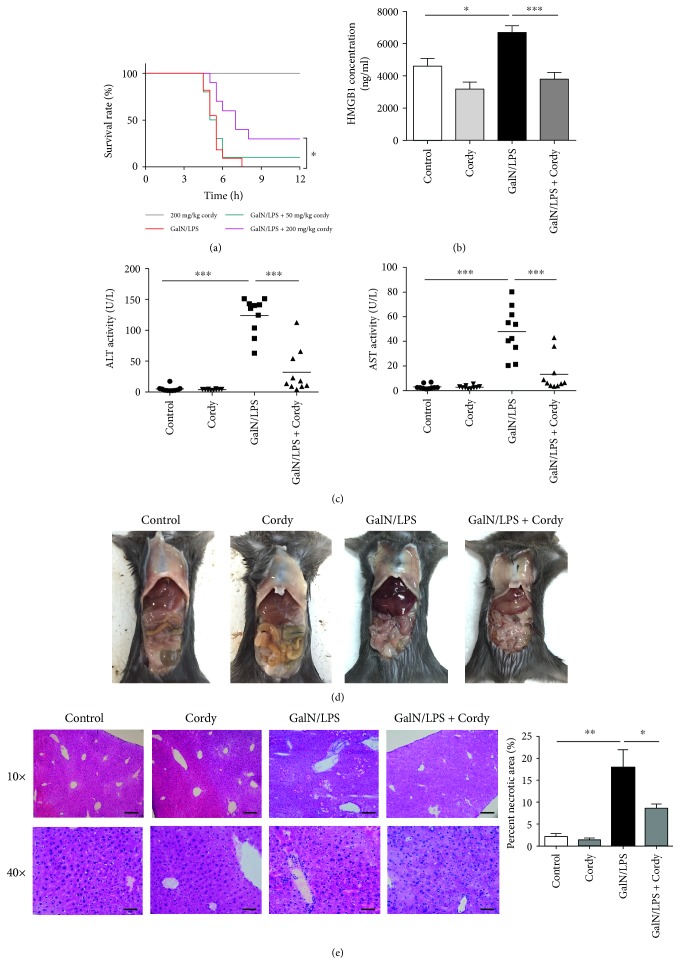
Cordycepin protects against hepatic damage induced by GalN/LPS. C57BL/6J mice were orally administrated with 50 mg/kg or 200 mg/kg cordycepin for 3 consecutive weeks, and GalN (700 mg/kg)/LPS (15 *μ*g/kg) was injected at 1 h before the last gavage. (a) The mortality rates were observed at various time points within 12 h after injected with a lethal dose of GalN/LPS (*n* = 10). The blood and liver tissues were harvested at 5 h after GalN/LPS treatment. (b and c) Then serum ALT, AST, and HMGB1 levels were assayed (*n* = 10). Represent visibly different damaged livers (d) and H&E staining analysis of liver sections (e) were shown. Upper scale bars: 100 *μ*m. Under scale bars: 50 *μ*m. Percent of necrotic area was calculated (*n* = 5). Each value is mean ± SEM. ^∗^*P* < 0.05, ^∗∗^*P* < 0.01, ^∗∗∗^*P* < 0.001.

In our study, all mice suffered severe liver injury and died within 12 h after injection with 700 mg/kg GalN plus 15 *μ*g/kg LPS. Compared with mice only treated with GalN/LPS, there was no markedly protective effect in mice administrated with 50 mg/kg cordycepin, but with a dose of 200 mg/kg cordycepin, the survival time significantly prolonged and the mortality markedly decreased approximately by 30% (Figure 1(a)). Meanwhile, the levels of plasma ALT, AST, and HMBG1, which indicate hepatic function and hepatocyte necrosis, were also determined at 5 h after GalN/LPS injection. Apparent increases in serum ALT, AST, and HMBG1 were observed in mice injected with GalN/LPS alone compared with control group, whereas cordycepin treatment at 200 mg/kg suppressed their growth (Figures 1(b) and 1(c)). From both macroscopic examination and histopathological inspection, GalN/LPS treatment caused many necrotic hepatocytes in centrilobular region, massive immune cell immigration to sinusoids, and hemorrhage, which were tremendously ameliorated by cordycepin administration at a dose of 200 mg/kg (Figures 1(d) and 1(e)).

**Figure 2:**
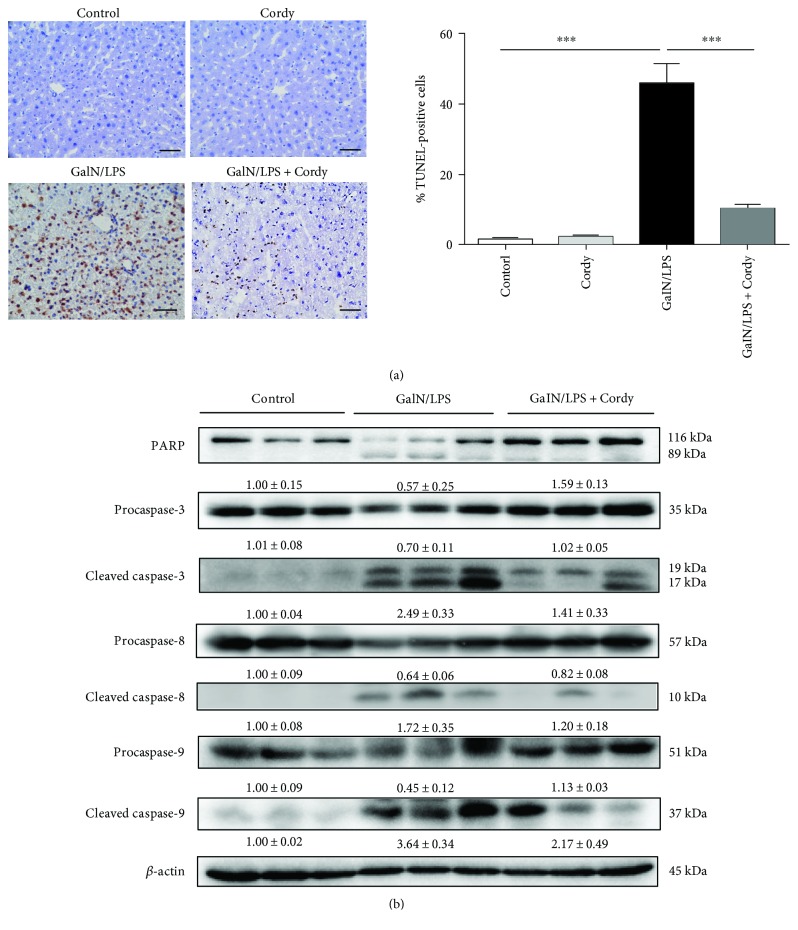
Cordycepin reduces hepatocyte apoptosis caused by GalN/LPS injection. Murine liver tissues were collected at 5 h after injected with GalN (700 mg/kg)/LPS (15 *μ*g/kg). (a) Hepatocyte apoptosis were measured by TUNEL staining, and the number of apoptotic cells were counted (*n* = 3). Scale bars: 50 *μ*m. (b) The level of cleaved caspase-3, caspase-8, and caspase-9 and PARP protein expressions were measured as indicated (*n* = 5). Each value is mean ± SEM. ^∗^*P* < 0.05, ^∗∗^*P* < 0.01, ^∗∗∗^*P* < 0.001.

As shown in Figure 2(a) using TUNEL method, the number of apoptotic cells greatly increased due to GalN/LPS injection (46.2 ± 11.8 per 100 nuclei), whereas pretreatment of 200 mg/kg cordycepin prevented hepatocyte apoptosis (10.4 ± 2.6 per 100 nuclei). In accordance with TUNEL data, the concentrations of cleaved caspase-8 and caspase-9, one mediated death receptor pathway and the other mediated mitochondrial intrinsic pathway, were upregulated; nevertheless, the expressions of full length ones were downregulated in GalN/LPS-treated group. Similar circumstances were present in both caspase-3 and PARP, which are effectors of apoptotic pathway; however, cordycepin (200 mg/kg) administration reversed these changes (Figure 2(b)). In cordycepin-alone treated group, no abnormal alterations were found in these indexes described above when compared with normal mice.

### 3.2. Cordycepin Inhibited Hepatic Inflammatory Cytokine Production and Inflammatory Cell Infiltration Induced by GalN/LPS

**Figure 3:**
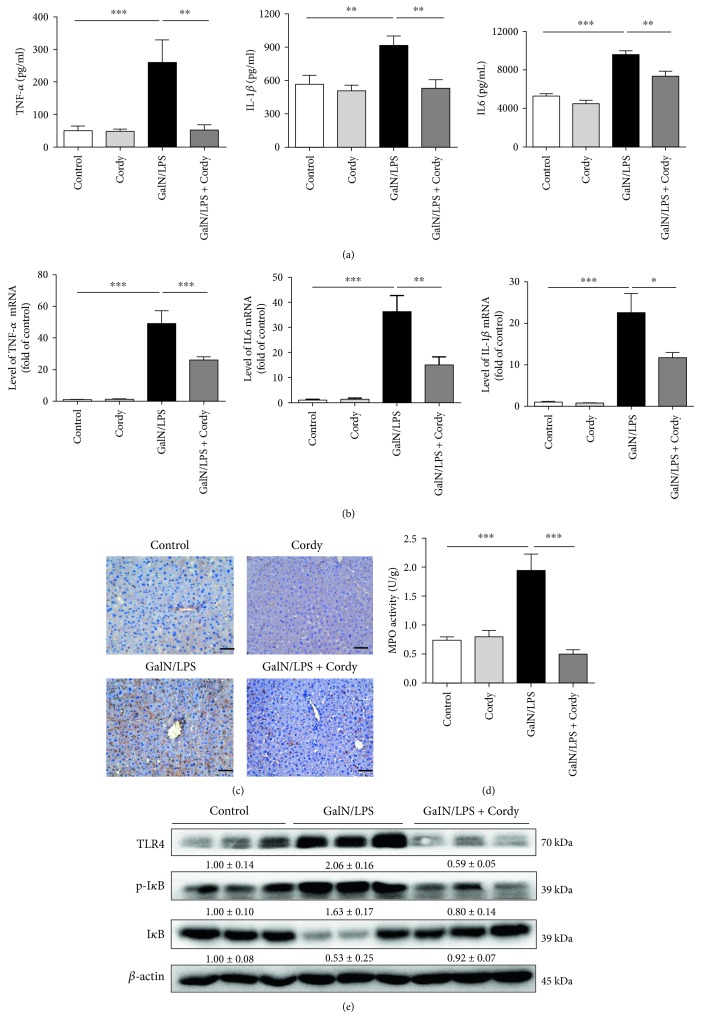
Cordycepin suppresses inflammatory cytokine production and attenuates TLR4 and NF*κ*B signal activation. Serum samples and liver tissues were harvested at 5 h after injected with GalN/LPS to mice. (a) Serum TNF-*α*, IL-1*β*, and IL-6 levels were assayed using ELISA method (*n* = 8). (b) Hepatic TNF-*α*, IL-1*β*, and IL-6 mRNA expressions were determined by qRT-PCR (*n* = 5). (c) F4/80 staining of liver sections were conducted to indicate the filtration of macrophages. Scale bars: 50 *μ*m. (d) Hepatic MPO activity was measured according to manufacturer's instruction (*n* = 8). (e) Hepatic expression of TLR4, I*κ*B, and p-I*κ*B was analyzed by western blotting (*n* = 5). Each value is mean ± SEM. ^∗^*P* < 0.05, ^∗∗^*P* < 0.01, ^∗∗∗^*P* < 0.001.

Since TNF-*α* and other inflammatory cytokines such as IL6 and IL-1*β* are important contributors to liver damage induced by GalN/LPS, we then assayed circulating TNF-*α*, IL6, and IL-1*β* levels at 5 h after GalN/LPS injection. As expected, GalN/LPS induced remarkable elevation of serum TNF-*α*, IL6, and IL-1*β* levels in contrast to those in the control groups, but pretreatment with 200 mg/kg cordycepin tremendously inhibited their production (Figure 3(a)). Similar results were obtained when measuring the mRNA expression of TNF-*α*, IL6, and IL-1*β* in liver tissues, demonstrating that cordycepin can inhibit GalN/LPS-induced inflammatory cytokine production through suppressing mRNA transcription (Figure 3(b)).

As Kupffer cells (hepatic macrophages) are the primary sources for these inflammatory cytokines when suffered with LPS challenge, we next conducted immunohistochemistry experiments with F4/80 antibody, which is a well-established marker of hepatic macrophages. As shown in Figure 3(c), there were massive inflammatory cell infiltration into liver tissues after GalN/LPS challenge while cordycepin (200 mg/kg) attenuated the increase of local macrophages. Equally, GalN/LPS induced an elevation of hepatic MPO activity, another sign for macrophage or neutrophil infiltration, which was also prevented by 200 mg/kg cordycepin (Figure 3(d)). Identically, the normal animals received with cordycepin had no significant changes when compared with the control group. Therefore, cordycepin was believed to exert hepatoprotective effect in animal model treated with GalN/LPS partly due to its inhibition of inflammatory response.

### 3.3. Cordycepin Blocked Hepatic TLR4 Expression and NF-*κ*B Activation Induced by GalN/LPS

It has been suggested that TLR4 and its downstream NF*κ*B signal pathway are mainly implicated in LPS-triggered macrophage inflammatory cytokine release [19]. I*κ*B was confirmed to be a critical inhibitor for NF*κ*B, and phosphorylation in Ser32 accelerated its degradation [20]. Therefore, the levels of I*κ*B and phosphor-I*κ*B reflect the activity of NF*κ*B indirectly. Then we test whether cordycepin could affect TLR4, I*κ*B, and phosphor-I*κ*B expression in vivo. The results of western blotting indicated that GalN/LPS-induced elevated expression of TLR4 and phosphorylated I*κ*B in hepatocytes were attenuated by pretreatment with 200 mg/kg cordycepin. Similarly, the reduced expression of hepatic I*κ*B by GalN/LPS injection was also reversed by 200 mg/kg cordycepin (Figure 3(e)), implying that cordycepin might exert its anti-inflammatory effect via decreasing TLR4 expression and inhibiting NF*κ*B activation in GalN/LPS-induced acute liver injury.

### 3.4. Cordycepin Inhibited Oxidative Stress Induced by GalN/LPS

**Figure 4:**
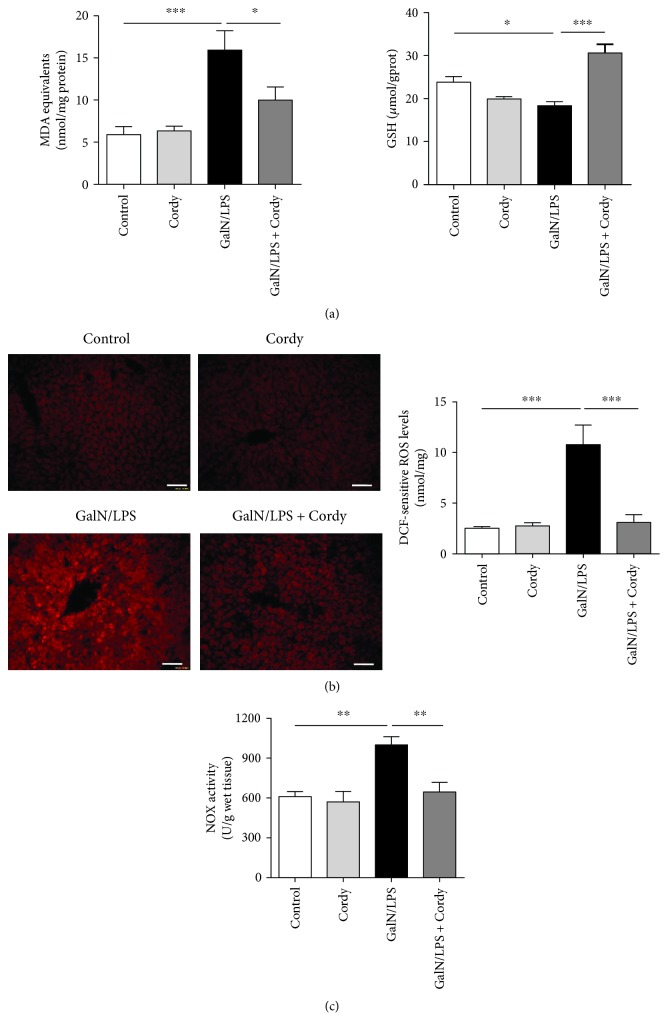
Cordycepin prevents oxidative stress induced by GalN/LPS. Murine liver tissues were collected at 5 h after injected with GalN (700 mg/kg)/LPS (15 *μ*g/kg). (a) Hepatic MDA and GSH levels were determined using commercial kits (*n* = 8). (b) The ROS levels in the liver tissues were visualized by DHE staining and measured by DCF assay (*n* = 8). Scale bars: 50 *μ*m. (c) Hepatic NOX activity was determined using NADH as substrates (*n* = 5). Each value is mean ± SEM. ^∗^*P* < 0.05, ^∗∗^*P* < 0.01, ^∗∗∗^*P* < 0.001.

During the initiation of liver damage, neutrophils or macrophages release large quantities of ROS by the phagocytic isoform of NOX complex [21]. To ascertain whether oxidative stress is involved in cordycepin's hepatoprotection against GalN/LPS-induced injury, the contents of lipid peroxidation product MDA, reductive product GSH, and ROS were determined. We observed significant accumulations of intracellular MDA and ROS as well as a reduction of GSH concentration in liver tissues of GalN/LPS-treated mice, which was reversed by the pretreatment with 200 mg/kg cordycepin (Figures 4(a) and 4(b)). Besides, a great impairment of enhanced NOX activity induced by GalN/LPS was found in the livers of mice pretreated with 200 mg/kg cordycepin (Figure 4(c)). Together, these data supported that cordycepin may improve oxidative stress in damaged liver, another potential explanation for its protection against GalN/LPS-induced hepatic injury.

### 3.5. Cordycepin Prevented Excessive Autophagy Induced by GalN/LPS

**Figure 5:**
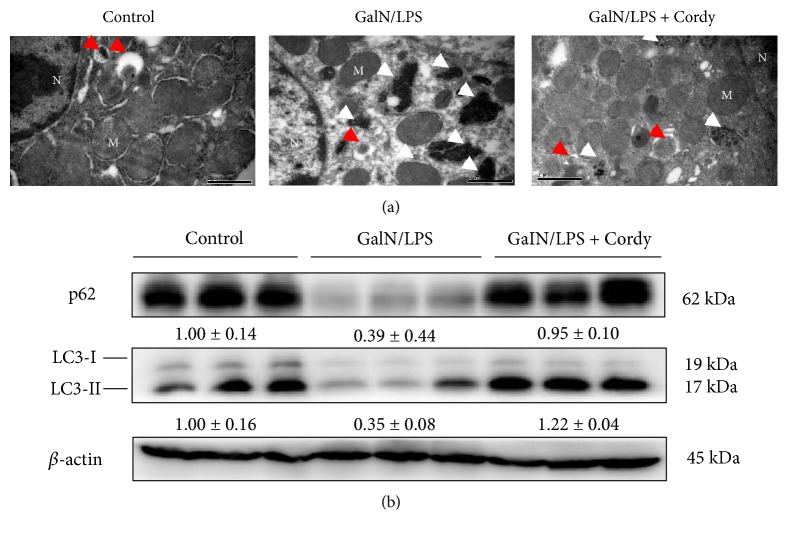
Cordycepin blocks excessive autophagy. Murine liver tissues were collected at 5 h after injected with GalN (700 mg/kg)/LPS (15 *μ*g/kg). (a) Electron microscope analysis of liver tissues. Red arrows denote autophagosomes. White arrows denote autolysosomes. N: nucleus; M: mitochondria. Bars: 1 *μ*m. (b) Immunoblot analysis of LC3 and p62 protein expressions (*n* = 5). Each value is mean ± SEM.

Mitochondria autophagy (also called mitophagy) is usually induced to self-remove dysfunctional mitochondria when suffered stress. However, excessive levels of autophagy are frequently associated with cell death [22]. From the results of electron macroscopy (Figure 5(a)), we could find electron-scarce cytoplasm and abundant late autophagic vacuoles (autolysosomes), characterized by single limiting membrane surrounding the electron-dense mitochondria debris, in hepatocytes from mice by GalN/LPS administration. Pretreatment with 200 mg/kg cordycepin significantly reduced the number of autolysosomes. LC3, a mammalian homolog of yeast Atg8, is known as a marker for macroautophagy. LC3-I is localized in cytoplasm while LC3-II (the phosphatidylethanolamine-conjugated form of LC3-I) exists in the membranes of the autophagosome. After fusion with the lysosome, LC3-II is degraded by lysosomal enzymes. Therefore, chronic activation of autophagic flux contributes to decreased expression of total LC3 [23]. Additionally, p62 (SQSTM1/sequestome 1), the autophagy substrate, is selectively degraded in autophagic flux [24]. Thus, p62 level is basically inversely correlated with autophagic degradation [25]. The western blot data showed decreased concentrations of p62 and total LC3 in liver tissues from model group whereas 200 mg/kg cordycepin treatment increased their expression (Figure 5(b)), demonstrating that cordycepin can prevent GalN/LPS-induced excessive autophagy.

## 4. Discussion

Mice injected with lethal dose of GalN/LPS to induce acute liver injury aims to replicate viral hepatitis-induced fulminant hepatic failure in clinic. Until now, it has become one of the most prevalent experimental murine models to investigate the potential therapeutic tools of fulminant hepatic failure. In the current study, we investigate the protective effects of cordycepin on acute liver injury induced by GalN/LPS. Specifically, pretreatment with cordycepin could raise the survival rate, improve liver function, decrease hepatocyte necrosis and apoptosis, and attenuate excessive autophagy, suggesting cordycepin may be an attractive therapeutic agent for viral hepatitis-induced fulminant hepatic failure.

Cordycepin, one of the primary bioactive components of *Cordyceps militaris*, has been reported to possess with anti-inflammatory and antioxidant activities. In LPS-stimulated macrophages and BV2 microglia, cordycepin significantly reduced the production of proinflammatory cytokines [12, 26]. In aged animal models, cordycepin treatment decreased oxidative damage and boosted the differentiation of bone marrow mesenchymal stem cells [27]. Emerging researches showed that abundant macrophage infiltration into the liver, producing excessive proinflammatory cytokines especially TNF-*α*, are the central steps to trigger hepatocyte apoptosis in GalN/LPS-induced acute liver injury [28]. For example, TNF-*α* or TNF-*α* receptor-deficient mice was able to resist the challenge of GalN/LPS [29]. In later stage, attracted neutrophils and monocytes by inflammatory cytokines and chemokines generate massive ROS and protease, contributing to hepatocyte necrosis, which is characterized by increased plasma HMGB1 released from dysfunctional tissues [28, 30]. Therefore, we speculated that administration with cordycepin might be profitable for acute liver injury caused by GalN/LPS injection. Indeed, our present results showed that cordycepin effectively antagonized GalN/LPS-induced elevated serum TNF-*α* levels, increased concentrations of hepatic ROS and oxidative product MDA, and decreased reductive product GSH, leading to alleviation of GalN/LPS causing impaired liver function (elevated ALT and AST) and massive hepatocyte apoptosis and necrosis, which is probably the reason why cordycepin could lower the high mortality rate induced by GalN/LPS.

To further explore the underlying mechanism by which cordycepin exerted anti-inflammatory effect, we assessed the expression of hepatic TNF-*α* mRNA, the activity of its leading transcriptional factor NF*κ*B, and the level of TLR4 protein. The canonical NF*κ*B signaling pathway is triggered once LPS interacts with its receptor TLR4 on the surface of hepatic macrophage [31]. Subsequently, I*κ*B, known as the inhibitor of NF*κ*B, is phosphorylated by upstream kinase IKK and sequential ubiquitinated which leads to its degradation. As expected, the current data demonstrated that cordycepin prevented the elevated expression of TLR4, depressed the phosphorylation of I*κ*B, and thus blocked GalN/LPS-induced NF*κ*B activation in the liver. The following elevated TNF-*α* mRNA level was also inhibited by administration with cordycepin, which may explain why there was lower concentration of plasma TNF-*α* compared to GalN/LPS treatment alone.

Enhanced MPO activity is the specific warning signal for neutrophil and macrophage infiltration, which is the dominant resource for ROS production in early stage [32]. Our data declared that cordycepin strikingly downregulated MPO activity, manifesting fewer hepatic neutrophils and macrophages, consistent with the result of F4/80 immunohistochemical stain, another biomarker for murine macrophage population [33]. Activated neutrophils and macrophages produce massive ROS, which will further incur hepatocyte necrosis. There are two main resources for ROS, one from NOX complex, which generates reactive oxygen using NAD(P)H as substrates. In the current study, reduced NOX activity was found in livers from mice pretreated with cordycepin when compared with mice injected with GalN/LPS, suggesting that cordycepin may improve oxidative stress via inhibiting NOX activity. Similar results were obtained in albumin-induced epithelial-mesenchymal transition of renal tubular cells [34]. However, whether there is direct suppressing effect on NOX complex by cordycepin remains unknown and further experiments are required.

Another principal source for ROS is the leakage from electron transfer chain in the mitochondria, especially the damaged ones. Massive superoxide release lead to sustained activation of c-Jun N-terminal kinase (JNK) and finally cause necrotic cell death [35]. Meanwhile, mitochondria autophagy (also called mitophagy) is induced to selectively eliminate dysfunctional ones, which seems to be protective. However, bulk mitophagy was also observed accompanying with great numbers of dying cells. It was reported that GalN/LPS could induce autophagic flux in dysfunctional liver [36], and we also found abundant autolysosomes present in liver tissues of mice suffered with GalN/LPS challenge. Pretreatment with cordycepin reduced excessive levels of autolysosomes and inhibited autophagic activity. But whether cordycepin is an inhibitor of autophagy or decreases autophagic activity indirectly through suppressing oxidative stress remains to be determined.

## 5. Conclusion

Pretreatment with cordycepin is beneficial for acute liver injury caused by GalN/LPS injection for its anti-inflammation and antioxidation property. The underlying mechanism might be associated with the decreased expression of TLR4, the inhibition of NF*κ*B signaling pathway, and the following depressed generation of TNF-*α*, contributing to reduced hepatocyte apoptosis. Decreased NOX activity and suppressed production of ROS help alleviate oxidative damage and reduce hepatocyte necrosis. Besides, cordycepin is also shown to prevent excessive autophagic activity induced by GalN/LPS.
